# Janus Kinase Inhibitors and Interstitial Lung Disease Associated With Pediatric Rheumatic Diseases: An Unexplored Field

**DOI:** 10.7759/cureus.50928

**Published:** 2023-12-21

**Authors:** Evdoxia Sapountzi, Lampros Fotis, Eleni Kotanidou, Liana Fidani, Assimina Galli-Tsinopoulou

**Affiliations:** 1 2nd Department of Pediatrics, AHEPA University General Hospital, Aristotle University of Thessaloniki, Thessaloniki, GRC; 2 Department of Pediatrics, Attikon General University Hospital, National and Kapodistrian University of Athens, Athens, GRC; 3 Department of Medical Biology Genetics, Aristotle University of Thessaloniki, Thessaloniki, GRC

**Keywords:** baricitinib, tofacitinib, janus kinase inhibitors, rheumatic diseases, interstitial lung disease, targeted synthetic dmards

## Abstract

Rheumatic diseases are often complicated by lung disease, commonly presenting as interstitial lung disease (ILD), with potentially detrimental consequences for patient survival. Although less frequent in pediatric patients, pulmonary involvement may be observed in almost all childhood-onset rheumatic conditions. The development of biological disease-modifying anti-rheumatic drugs has significantly improved clinical outcomes. However, disease remission is not always complete or long-lasting, and treatment may need to be discontinued due to adverse effects. A novel class of drugs, namely Janus kinase inhibitors (JAKis), has been proposed to provide a significant survival benefit for patients with rheumatic diseases. Despite the ample literature on the efficacy and safety of JAKis in rheumatic disease, only a few studies have investigated the effectiveness of these drugs in patients with pulmonary involvement, and only two case reports have presented results in pediatric patients. We provide an overview of the rationale for using JAKis in ILDs associated with rheumatic disease and summarize the main studies evaluating their efficacy in both adult and pediatric patients. The present review highlights the need for controlled long-term studies to assess the efficacy and safety of JAKis in pediatric rheumatic disease complicated by lung disease.

## Introduction and background

Pediatric rheumatic diseases encompass a group of disorders that range from musculoskeletal, autoinflammatory, and vasculitic diseases to connective tissue disorders with an onset in childhood. It is estimated that approximately 6-7 million children worldwide suffer from rheumatic diseases [1]. The most common rheumatic disease involving the musculoskeletal system among pediatric patients is juvenile idiopathic arthritis (JIA). Other common pediatric rheumatic diseases include Henoch-Schonlein purpura (a pediatric vasculitis), systemic lupus erythematosus (SLE), acute rheumatic fever, Kawasaki disease, and juvenile dermatomyositis (JDM) [2,3].

Although uncommon, pulmonary involvement may be observed in almost all pediatric rheumatic conditions [4]. In adult patients with various types of rheumatic diseases, interstitial lung disease (ILD) is the primary manifestation of lung involvement, causing progressive respiratory failure that significantly impairs the patient’s quality of life and increases their morbidity and mortality [5,6]. The incidence of lung manifestations among pediatric patients with rheumatic conditions varies considerably. While more than 50% of patients with juvenile-onset systemic sclerosis develop ILD, the prevalence of lung disease in patients with JIA is estimated to be between 4% and 8% [7-10] and approximately 6.8% in patients with systemic JIA (SJIA), representing 10-20% of the JIA cases [11]. Several studies have focused on ILD associated with rheumatic disease in adults; however, research in pediatric patients is limited.

The treatment options for rheumatic diseases have been revolutionized by the development of biological disease-modifying anti-rheumatic drugs (DMARDs), which have significantly improved clinical outcomes. Nevertheless, some patients show no or only a partial response to these therapies or experience adverse effects [12]. Janus kinase inhibitors (JAKis) are small molecule inhibitors of the Janus kinase family of receptors classified as targeted synthetic DMARDs. Several JAK pathways have been implicated in the pathogenesis of rheumatic diseases such as rheumatoid arthritis (RA) [13] and ILD [14]. Currently approved JAKis for adult patients with RA include tofacitinib, baricitinib, peficitinib, upadacitinib, and filgotinib, which have shown a good safety profile [15]. Tofacitinib and baricitinib were also approved for JIA for children over two years old [16,17]. Clinical trials are ongoing for JAKis, especially tofacitinib, for patients with rheumatic disease, including SLE, type 1 interferonopathies, JDM, SJIA, and polyarticular JIA groups [18-20]. JAKis have been shown to be effective for the treatment of RA, either as monotherapy or in combination with methotrexate; however, there is limited literature on their efficacy, safety, and tolerability in patients with RA-associated ILD [21], and information is even more scarce for pediatric patients.

This review provides an overview of the rationale for using JAKis in ILD associated with rheumatic disease and summarizes the main studies evaluating their efficacy in both adult and pediatric patients.

## Review

Methods

We searched scientific platforms such as PubMed, Scopus, and Google Scholar for articles related to JAKis and the treatment of rheumatic diseases in adult and pediatric patients with ILD. The following search words were used: JAK inhibitor, tofacitinib, baricitinib, peficitinib, upadacitinib, filgotinib, rheumatic disease, juvenile idiopathic arthritis, rheumatic arthritis, juvenile dermatomyositis, systemic lupus erythematosus, lung disease, ILD, lung involvement, pediatric, children, juvenile, and adult. Relevant articles for the reference sections of the screened articles that could be consistent with the subject of the review were also included. The search was limited to studies published in the English language. Otherwise, all types of articles were considered, including reviews, research articles, case reports, and conference abstracts.

ILD in rheumatic diseases

ILD is the most serious complication associated with systemic rheumatic diseases, leading to significant morbidity and mortality [22]. In RA, the prevalence of ILD is approximately 10%, although rates as high as 67% have been reported depending on the method used to diagnose ILD (e.g., CT versus high-resolution CT (HRCT)) [23]. While RA-ILD appears to predominantly affect male patients [24], a study in pediatric patients showed that lung involvement in rheumatic disease is more strongly associated with female sex [4].

ILD is reported as one of the leading causes of death among adult patients with RA alongside malignancy, cardiovascular disease, and infection [25]. The median patient survival is 2.6 years, compared to 9.9 years in patients with RA but without ILD, and the standardized mortality ratio for RA-ILD compared with RA without ILD is 2.86 [26]. The most common patterns of ILD described in RA are usual interstitial pneumonia and nonspecific interstitial pneumonia [27]. Common symptoms include a dry cough, shortness of breath, and fatigue [28].

Despite the availability of information in adults, reports regarding lung involvement in children with rheumatic diseases are scarce. The overall occurrence of lung involvement in pediatric rheumatic diseases is rare; however, an increasing incidence of ILD in pediatric patients with SJIA has been demonstrated since 2010, with a prevalence in 2020 estimated at nearly 7% [29]. The onset of the disease in these patients is typically before two years of age, while ILD usually appears approximately 1.5 years after the initial presentation of SJIA [29]. Common clinical features include minimal respiratory symptoms, lymphadenopathy, hepatosplenomegaly, and clubbing [29]. Nevertheless, these children are often clinically asymptomatic until the disease is significantly advanced [30]. Indeed, Huang et al. [4] showed a relatively high prevalence of lung involvement in pediatric patients with newly diagnosed rheumatic diseases, the majority of whom had abnormal pulmonary function tests and HRCT findings, even though most were asymptomatic. The most common finding in these patients was a reduction in lung diffusion for carbon monoxide values, a prominent marker of ILD [31]. In their study, Huang et al. [32] showed that the majority of patients with SJIA and ILD (84%) were carriers of the HLA-DRB1*15 haplotype, a percentage that was significantly different from that of patients without ILD. Moreover, the levels of serum IL-18 and MMP-7 were much elevated in these patients, whereas variable levels of CXCL9 were detected [32].

The pathophysiology of lung involvement in adult rheumatic diseases arises due to the combined interplay of various genetic, environmental, and autoimmune factors that induce an exacerbated tissue response in the alveolar wall and pulmonary parenchyma [33]. ILD is characterized by varying degrees of inflammation or fibrosis in the pulmonary parenchyma. However, the exact mechanism of ILD development is not well understood. In adults, the long-term presence of specific serum circulating antibodies is thought to cause a series of events, particularly when genetic and/or environmental predispositions exist, leading to an inflammatory response that results in the manifested disease [33]. In children, other factors may contribute to the disease, although the involved mechanism remains elusive [11].

Rationale for targeting the JAK pathway in ILD

The inflammatory process that results in ILD involves the activation of cytokines, chemokines, and growth factors, such as tumor necrosis factor (TNF), vascular endothelial growth factor (VEGF), fibroblast growth factor (FGF), platelet-derived growth factor (PDGF), and interleukins (IL). These mediate fibroblast proliferation and differentiation, increase the synthesis and deposition of the extracellular matrix, and enhance the activity of matrix enzymes [13]. Gene expression profiling of lung tissue in patients with SJIA-related ILD revealed interferon-γ (IFN-γ) pathway activation [34]. Moreover, the levels of serum IL-18 and MMP-7 were much elevated in these patients, whereas variable levels of CXCL9 were also detected [30]. In patients with systemic sclerosis-related ILD, plasma levels of TNFα and IL-6 were elevated [35,36].

All the above-mentioned factors can activate the JAK/STAT molecular pathway, initiating cellular changes observed in ILD [13]. In addition to the inflammatory response leading to JAK/STAT activation, several members of the JAK/STAT pathway are overexpressed in ILD, justifying the targeting of this pathway in therapeutic strategies. Kubo et al. showed the different JAK/STAT signaling pathways activated in ILD [13].

JAKis

The JAK-STAT pathway regulates the transcription of several genes involved in inflammatory, immune, and cancer conditions. Hence, it is not surprising that targeting the JAK family of kinases with small molecule inhibitors is effective for the treatment of various diseases [37]. However, JAK inhibition is a complex matter, considering the large number of molecules that activate this pathway. JAK proteins, including JAK1, JAK2, JAK3, and TYK2, mediate the signaling of more than 40 cytokines. This means that JAK inhibition will block the signaling of multiple cytokines simultaneously [38]. Therefore, the specificity of available drugs and those under development plays a crucial role in the treatment outcome and is related to potential adverse events.

Compared to biological DMARDs, which are large molecules administered parenterally, JAKis are orally available, which makes them preferable for treatment. JAKis share a common mechanism of action that involves blocking the enzyme’s ATP binding site and hence blocking downstream signaling [39]. Although JAKs are structurally similar, the ATP binding site, critical to their function, is subtly different among different JAKs, which has enabled the development of relatively selective JAKis [40]. Hu et al. explained JAKs and their differences in terms of expression and downstream signaling [41].

According to their selectivity against different JAK molecules, JAKis are divided into first and second generations. The first generation includes non-selective inhibitors and the second generation comprises more selective inhibitors that block fewer cytokines. Of the 11 JAKis approved for clinical use to date, five have been marketed for the treatment of RA in adults: tofacitinib and baricitinib, constituting first-generation JAKis, and peficitinib, upadacitinib, and filgotinib, belonging to the second-generation JAKis (Table 1). Since 2020, tofacitinib has also been approved by the FDA for polyarticular JIA [42], while baricitinib was investigated in a phase III trial for patients with extended oligo- or polyarticular JIA, with promising results in terms of efficacy and safety [17]. Moreover, the FDA has issued an emergency use authorization to allow the emergency use of baricitinib for the treatment of coronavirus disease in 2019 in hospitalized pediatric patients aged 2-18 years who require supplemental oxygen, mechanical ventilation, or extracorporeal membrane oxygenation [43].

**Table 1:** Marketed JAKis for the treatment of RA AA: alopecia areata; AD: atopic dermatitis; AS: ankylosing spondylitis; CD: Crohn's disease; COVID-19: coronavirus disease; nr-axSpA: non-radiographic axial spondyloarthritis; pJIA: polyarticular course juvenile idiopathic arthritis; PsA: psoriatic arthritis; RA: rheumatoid arthritis; UC: ulcerative colitis, FDA: Food and Drug Administration, JAK: Janus kinase

Marketed JAKis	First approval date	FDA-approved indications other than RA	Countries of approval	JAK selectivity
Tofacitinib	2012	AS, PsA, UC, pJIA	>80 countries, including USA, EU countries, Korea	Mainly JAK1 and JAK3, but also JAK2
Baricitinib	2017	AA, COVID-19	>75 countries, including EU countries, USA	JAK1, JAK2
Peficitinib	2019	Drug not approved by FDA	Japan, South Korea, and Taiwan	Mainly JAK3, but also other JAKs
Upadacitinib	2019	AS, PsA, nr-axSpA UC, CD, AD	USA, EU, UK	Mainly JAK1, also JAK2
Filgotinib	2020	Drug not approved by FDA	EU, Japan	Mainly JAK1

Efficacy of JAKis in ILD associated with rheumatic disease

The efficacy and safety of JAKis in rheumatic diseases, mainly RA in adults, as well as in RA-associated ILD, have been demonstrated in several clinical trials but also in real-world studies [21,44-48]. A potential role for JAKis has also been proposed for the treatment of systemic sclerosis-associated ILD [49]. Focusing on RA-ILD, a multicenter observational study by Kalyoncu et al. [43] included 47 patients with RA who also had ILD based on HRCT findings and were treated with tofacitinib. Most patients showed stable pulmonary functions after one year of treatment, while only a few patients had to change treatment due to a worsening in pulmonary functions. Tardella et al. [21] retrospectively analyzed 31 patients with RA-associated ILD, of whom 18 received baricitinib and 13 received tofacitinib. More than 80% of patients showed stable lung involvement or improvement after 18 months of treatment, as determined by HRCT. Another retrospective study by Venerito et al. [47] included 43 patients with RA-ILD treated with JAKis: 28 with baricitinib, nine with tofacitinib, and three with filgotinib or upadacitinib. In addition to JAKis, 16 patients also received methotrexate. After at least six months of active therapy, forced vital capacity remained stable or improved in >80% of patients; diffusing lung capacity for carbon monoxide (DLCO) showed a similar trend; and HRCT findings remained stable or improved in around 90% of the patients.

Since ILD occurrence has been associated with the systemic or prolonged use of biologics, Mochizuki et al. [50] analyzed 36 patients with RA-ILD treated with tofacitinib to determine the rate of pulmonary toxicity. The authors found very low rates of lung function deterioration after one year of treatment, concluding that the use of JAKis is safe and not associated with pulmonary toxicity. Such a risk of toxicity was also excluded for baricitinib in an analysis of eight studies and 3770 patients with RA [51].

In contrast to the availability of data in adults, the efficacy of JAKis in pediatric patients with rheumatic disease has not been extensively investigated, particularly in the context of JIA. Ruperto et al. [16] conducted a double-blind, placebo-controlled, randomized phase 3 withdrawal trial including 142 patients with polyarticular JIA, treated with tofacitinib (n=72) or placebo. Patients receiving tofacitinib showed a lower flare rate and a significantly longer time to the presentation of flares, with 71% remaining flare-free compared with those receiving placebo. The authors reported similar safety findings in both groups. Similar promising findings were reported for baricitinib in the respective ongoing phase 3 trial [17]. Rahman et al. [52] followed 27 patients with refractory polyarticular JIA who received tofacitinib for up to 24 weeks. Treatment led to a significant reduction in the disease activity score, with only minimal side effects reported. Kostik et al. [42] evaluated 24 patients with different rheumatic diseases (15 JIA, seven undifferentiated systemic autoinflammatory diseases, and two JDM). After treatment with tofacitinib for at least six months, 12 patients with JIA and all patients with JDM or undifferentiated systemic autoinflammatory disease showed a complete or partial response, suggesting the efficacy of tofacitinib in these immune-mediated diseases.

Regarding ILD associated with pediatric rheumatic disease, only a few children have been reported to date to have received JAKis [53-58]. Basile et al. [53] reported the case of a two-year-old girl who presented with intermittent limping and joint pain, had a family history of Still’s disease (SJIA) with lung involvement, and was positive for rheumatoid factor, antinuclear antibodies, and cyclic citrullinated peptide. A CT examination revealed severe interstitial pneumopathy with diffuse fibrosis. Genetic testing also revealed a mutation in the COPA gene, and thus the diagnosis of COPA syndrome was made. The girl was treated with baricitinib, and after two weeks, her limping and joint pain improved dramatically. Unfortunately, the authors did not perform a repeat CT after treatment to assess the pulmonary outcome. Bader-Meunier et al. [54] reported the case of a two-year-old girl with refractory SJIA and associated ILD. The patient received corticosteroids, which, although efficient at first, did not achieve sustained remission. Treatment was continued with several lines of biological agents, none of which were effective or tolerated. At the age of four years, the patient was treated with ruxolitinib, a JAK1/2 inhibitor currently approved for vitiligo but not for rheumatic diseases. After 15 months of treatment, there was an improvement in chest CT findings as well as an increase in oxygen saturation [54]. Sabbagh et al. [55] reported two patients with anti-MDA5 autoantibody-positive JDM and lung involvement (12 and 15 years old, respectively) who showed improvement in DLCO after treatment with tofacitinib. Specifically, DLCO improved from 56% to 96% predicted value in the first patient and from 41% to 65% in the second after six months of tofacitinib treatment. Yu et al. [56] also reported two patients with JDM complicated with ILD: an 11-year-old Chinese female positive for anti-MDA5 antibodies and ILD and a 10-year-old female positive for antinuclear antibodies, anti-Mi-2β, and anti-Ro-52. Both patients were treated with 5 mg tofacitinib and showed improvement in DLCO after three months, from 52.4% to 59.6% predicted value and from 39.2% to 60% predicted value, respectively. Two more studies reported on patients with refractory JDM-ILD treated with tofacitinib; however, the treatment outcomes with regard to ILD were not reported [57,58].

## Conclusions

Although no definite conclusions can be drawn from the limited available studies exploring JAKis in pediatric patients, evidence from adult patients with rheumatic disease-associated ILD supports the use of JAKis as a valid therapeutic option for these patients. This review highlights the necessity for controlled prospective studies demonstrating the efficacy of JAKis in children. Moreover, as the long-term effects of JAKis are currently unknown, especially in children, it is essential to conduct long-term studies to carefully evaluate the benefit/risk balance before starting treatment.
